# Evaluation of Structural and Electrochemical Properties of Supercapacitors with Graphene Electrodes and Hydrated Pure or Mixed [bmim]-Based Ionic Liquids via Molecular Dynamics

**DOI:** 10.1021/acsphyschemau.5c00036

**Published:** 2025-07-15

**Authors:** Lucas de S. Silva, Guilherme Colherinhas

**Affiliations:** Instituto de Física, 67824 Universidade Federal de Goiás, 74690-900 Goiânia, GO, Brazil

**Keywords:** supercapacitors, hydrated ionic liquids, mixed ionic liquids, molecular dynamics, energy storage

## Abstract

This study investigates the effect of anion composition on the performance of supercapacitors (SCs) using hydrated ionic liquids and graphene electrodes, focusing on comparing pure and mixed electrolytes. Systems containing [bmim] paired with NO_3_^–^, ClO_4_^–^, and Br^–^ were evaluated to assess their impact on electric double layer (EDL) formation and electrochemical behavior. Molecular dynamics (MD) simulations were performed under varying surface polarization, focusing on interaction energies, species distribution, capacitance, and projected energy density. Capacitance values ranged from 2.30 to 2.55 μF/cm^2^, while energy densities varied between 5.03 and 5.58 J/g, depending on electrolyte composition. The results show that small, mobile anions like Br^–^ promote more compact EDLs and higher capacitance, even with weak electrode interactions. NO_3_^–^ contributes to interfacial organization through hydrogen bonding with water. Mixed anion systems demonstrated competitive performance, with the best results obtained by combining high ion mobility and structural organization. This suggests that hybrid electrolytes are a promising strategy for optimizing energy storage in ionic liquid-based SCs.

## Graphical abstract

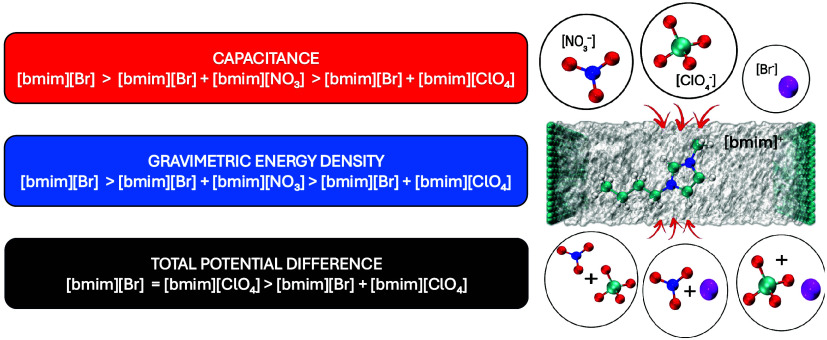

## 1 Introduction

The growing demand for efficient and rapid-response energy storage systems has driven the development of high-performance electrochemical devices. Among them, supercapacitors (SCs) have gained prominence due to their high-power density, long cycle life, and ultrafast fast charge and discharge times.^1–4^ These features make SCs ideal for applications requiring rapid energy delivery or recovery, such as electric vehicles, renewable energy systems, and portable electronic devices.^4,5^ However, fully exploit their capabilities, it is essential to optimize all components of the device, especially the electrodes and electrolytes, since energy storage in SCs occurs through the formation of the electric double layer (EDL), which results from ion adsorption of the electrolyte onto the electrode surfaces.

Among the most promising materials for SC electrodes, graphene stands out due to its exceptionally high surface area, excellent electrical conductivity, and remarkable chemical stability.^6–10^ These properties enable efficient charge storage via EDL formation at the electrode/electrolyte interfaces in SCs.^11,12^ Furthermore, its two-dimensional structure and the potential for surface functionalization allow for the modulation of interactions with the electrolyte, making graphene an ideal candidate for high-performance SC development.

On the other hand, room-temperature ionic liquids (RTILs) have been widely investigated as alternative electrolytes for SCs due to their broad electrochemical stability window, high thermal stability, and low volatility.^13–16^ However, pure RTILs exhibit limitations such as high viscosity and relatively low ionic conductivity,^17^ which can hinder ion transport within the system. A promising strategy to overcome these limitations is the systematic addition of water to RTILs, promoting electrolyte hydration. In our previous work,^18^ we demonstrated that the presence of water in the electrolyte can significantly enhance electrochemical performance by influencing the structure of the EDL. Specifically, we found that electrolyte hydration can significantly enhance both the capacitance and the gravimetric energy density of the devices. Another relevant approach to electrolyte optimization is the use of RTILs mixtures, combining different cations and anions in a single formulation.^19–22^ This strategy enables the exploitation of synergistic interactions among the mixture components, allowing precise modulation of properties such as viscosity, density, ionic conductivity, and solvation structure.^19–22^ Moreover, it offers greater flexibility in the design of customized electrolytes for specific applications, enhancing compatibility with electrode materials and extending the system’s electrochemical operating window.^19–22^

For instance, Ghahari and Raissi^23^ employed classical molecular dynamics (MD) simulations to investigate the relationships between interfacial interactions and the nanostructuring of imidazolium-based room-temperature ionic liquids (RTILs) around graphene electrodes, specifically focusing on the 1-ethyl-3-methylimidazolium ([emim]^+^) cation combined with NO_3_^–^, Cl^–^, BF_4_^–^, and SCN^–^ anions. The authors found that the imidazolium cation adopts a parallel orientation relative to the graphene surface due to π–π interactions, leading to the formation of a highly ordered interfacial layer. They also showed that different anions markedly influence the structure of the EDL and the ionic dynamics, thereby affecting both the capacitance and the stored energy. These findings highlight the potential of jointly tuning the electrolyte composition and electrode structure to enhance supercapacitor performance. On the other hand, Oliveira et al.^24^ explored how the addition of small-sized ions, specifically Br^–^ and Cl^–^, affects the composition of hydrated electrolytes in supercapacitors based on 1-butyl-3-methylimidazolium ([bmim]^+^), using molecular dynamics simulations. The authors demonstrated that increasing the concentration of chloride ions in water-containing IL electrolytes significantly enhances the gravimetric energy density of graphene-based supercapacitors, yielding an improvement of approximately 10%, despite causing negligible changes in capacitance or charge distribution. They also observed that water preferentially accumulates near the positive electrode, following the anionic distribution, and that water–electrode interactions — especially those mediated by anions — are critical to the interfacial structuring of the electrolyte. Furthermore, it was shown that higher hydration levels improve gravimetric efficiency, although viscosity, even when reduced in hydrated media, remains an important limiting factor for practical applications.

Salisu et al.^25^ proposed an innovative approach involving a device composed of a microemulsion-based electrolyte combined with graphene electrodes. They reported a wide electrochemical voltage window ranging from 2.2 to 2.4 V, with specific capacitances of 59 F/g at 0.1 A/g and 32 F/g at 5 A/g. This type of electrolyte consists of a thermodynamically stable mixture of two immiscible liquids stabilized by surfactants, highlighting that the use of liquid mixtures in electrolytes can be a promising strategy for enhancing supercapacitor performance. Schütter et al.^26^ investigated the use of binary mixtures of the ionic liquid [Pyr_14_]­[TFSI] with organic solvents in supercapacitors, aiming to evaluate transport properties and electrochemical performance in devices employing activated carbon electrodes. The study revealed that these mixtures exhibited high electrochemical stability, withstanding voltages of up to 3.2 V, further supporting the notion that the use of mixed electrolytes is a beneficial strategy for electric double-layer capacitors. Lian et al.^27^ investigated the effect of IL mixture composition on the structure of the EDL and the capacitance of SCs. In this study, the authors focused on electrolytes based on [emim]­[TFSI] and [emim]­[BF_4_] and found that combining these ILs at a specific ratio increases the counterion density within the EDL, leading to enhanced capacitance. Moreover, the study revealed good agreement between theoretical simulations and experimental data, confirming the potential of using ionic liquid mixtures as an effective strategy to optimize supercapacitor performance.

In this context, the present study investigates the structural and electrical properties of SCs composed of graphene electrodes and electrolytes made of individual hydrated RTILs and their mixtures. Using classical full atomistic MD simulations, we analyzed combinations of RTILs based on the 1-butyl-3-methylimidazolium ([bmim]) cation with bromide, nitrate, and perchlorate anions, specifically: (Model-1) [bmim]­[NO_3_] + H_2_O; (Model-2) [bmim]­[ClO_4_] + H_2_O; (Model-3) [bmim]­[Br] + H_2_O; (Model-4) [bmim]­[ClO_4_] + [bmim]­[Br] + H_2_O; (Model-5) [bmim]­[ClO_4_] + [bmim]­[NO_3_] + H_2_O; and (Model-6) [bmim]­[Br] + [bmim]­[NO_3_] + H_2_O. The aim of this study is to understand how changes in electrolyte composition affect EDL formation and, consequently, the electrochemical performance of SCs. To this end, this paper is organized into four main sections: the next section presents the computational methodology, followed by the results and discussion, and finally, the conclusions. The models studied and the molecules that make up the electrolytes are highlighted in Figure 1.

**Figure 1:**
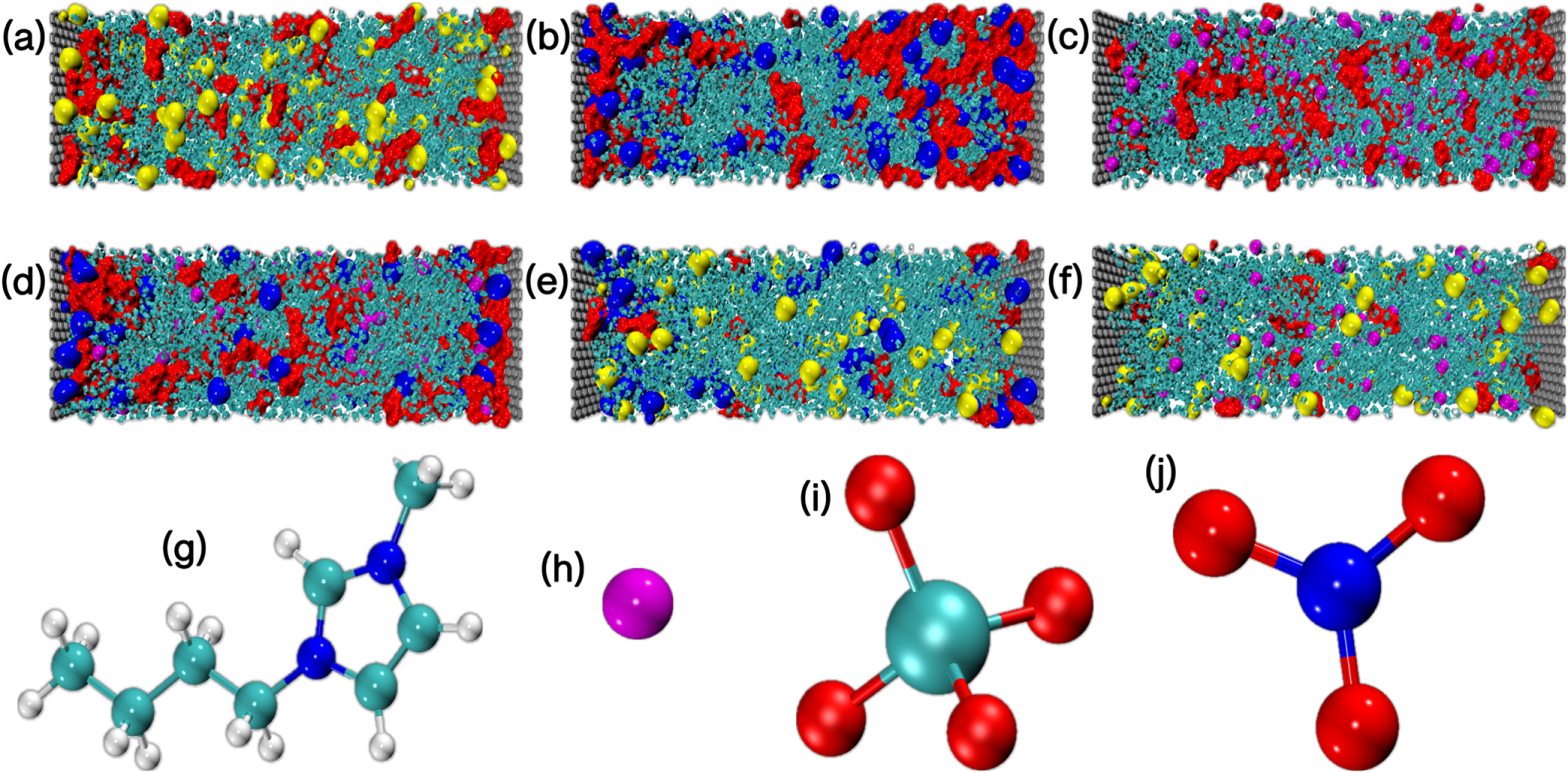
Visual representation of the systems investigated in this work. The highlighted configurations follow the order: (a) M1; (b) M2; (c) M3; (d) M4; (e) M5; (f) M6. Panels (g–j) show the molecular structures of the electrolyte components: (g) [bmim]^+^; (h) Br^–^; (i) ClO_4_^–^; and (j*)* NO_3_^–^. [bmim] molecules are represented in red, while NO_3_^–^, ClO_4_^–^ and Br^–^ are highlighted in yellow, blue and pink, respectively. Water molecules are represented in cyan.

## 2 Methods

To conduct this study, fully atomistic Molecular Dynamics (MD) simulations were employed to investigate the structural and energetic properties of SCs composed of graphene electrodes (with dimensions *X* = 3.708 nm, *Y* = 3.8529 nm) and electrolytes based on hydrated mixtures of RTILs, with the electrolyte concentration fixed at 2 M. Each electrode is modeled as a single-layer graphene sheet containing 540 carbon atoms, positioned at the left and right ends of the electrolyte limit to represent the negative and positive electrodes of the SC, respectively. The simulation box has a total length of 60 nm along the *z*-axis, with the two graphene electrodes placed 12 nm apart. The confined electrolyte is located between the electrodes, and its center is aligned with the center of the simulation box. The regions beyond the graphene electrodes are filled with vacuum, which serves to eliminate spurious interactions between the electrodes and their periodic images due to the periodic boundary conditions applied along the *z*-direction. To ensure the structural stability of the electrodes throughout the simulation and to prevent any artificial deformation or displacement caused by pressure or interactions with the ionic species, all carbon atoms in the graphene sheets are kept fixed. This constraint not only stabilizes the system but also simplifies the interpretation of the electrostatic potential profiles and ensures a well-defined and constant volume for the supercapacitor. For electrolytes, we focused on RTILs based on the 1-butyl-3-methylimidazolium ([bmim]) cation, combined with bromide, nitrate, and perchlorate anions, which are well established in the literature for energy storage applications in SCs.^23,28–32^ The systems studied are denoted as follows: (M1) [bmim]­[NO_3_] + H_2_O; (M2) [bmim]­[ClO_4_] + H_2_O; (M3) [bmim]­[Br] + H_2_O; (M4) [bmim]­[ClO_4_] + [bmim]­[Br] + H_2_O; (M5) [bmim]­[ClO_4_] + [bmim]­[NO_3_] + H_2_O; and (M6) [bmim]­[Br] + [bmim]­[NO_3_] + H_2_O. The detailed molecular composition of each system is presented in Table 1 and a schematic representation of the models and electrolyte constituent is shown in Figure 1. To ensure consistency across the systems, the number of ion pairs and water molecules was carefully adjusted to maintain a fixed molar concentration of 2 M in all simulations. As detailed in Table 1, each system contains a slightly different number of [bmim] cations, anions, and water molecules, with total masses and atom counts very similar across models. This approach allows for a fair and meaningful comparison of the structural and energetic properties of the different electrolyte compositions while preserving comparable solvent-to-ionic liquid ratios, as indicated by the consistent H_2_O/*RT*IL molar ratios shown in Table 1.

**Table 1:** Composition of the Systems Analyzed In This Study^a^

electrolyte (Models)	# [bmim] molecules	# A_1_ molecules	# A_2_ molecules	# H_2_O molecules	total mass (×10^–19^ g)	# atoms	H_2_O/RTLI
traditional-RTILs systems (2 M in water solution)							
M1 (A_1_=[NO_3_])	152	152	0	4219	1.98	18,145	27.8
M2 (A_1_=[ClO_4_])	148	148	0	4107	2.03	17,841	27.8
M3 (A_1_=[Br])	153	153	0	4246	2.04	17,796	27.8
mixed-RTILs systems (2 M in water solution)							
M4 (A_1_=[ClO_4_]; A_2_=[Br])	150	75	75	4184	2.04	17,832	27.9
M5 (A_1_=[ClO_4_]; A_2_=[NO_3_])	148	74	74	4175	2.00	17,971	28.2
M6 (A_1_=[Br]; A_2_=[NO_3_])	152	76	76	4239	2.01	17,977	27.9

a The table presents the number of each species (A_1_ and A_2_ represent the anions in the ionic liquid mixtures), the number of water molecules, the total mass of the system (electrolyte and electrodes), and the total number of atoms (electrolyte and electrodes). Additionally, it shows the ratio between the number of water molecules and the number of ionic liquid ion pairs. In all cases, the electrolyte concentration was kept constant at ∼2 M.

We emphasize that the selection of the nitrate (NO_3_^–^), perchlorate (ClO_4_^–^), and bromide (Br^–^) anions was motivated by their distinct physicochemical properties and hydration behaviors, which are known to influence the formation and structure of the EDL at the electrode–electrolyte interface. These differences can affect the electrochemical performance of SC. Moreover, maintaining a consistent ion concentration across the systems allows for the design of ionic mixtures with slightly varied total masses, aiming to optimize energy efficiency without compromising the electrochemical stability window of the device. Additionally, some of these anions exhibit stronger specific interactions with graphene or possess the ability to form hydrogen bonds with water molecules, which can impact the electrolyte structuring near the electrode surfaces and thus alter device behavior.

All systems were constructed using the PACKMOL software.^33^ The graphene electrodes were maintained at a fixed separation of 12 nm to simulate the conditions of a confined supercapacitor. To minimize undesired interactions caused by periodic boundary conditions, vacuum slabs of 24 nm were added beyond each electrode surface. These vacuum slabs ensure that interactions between the cell’s edges do not influence the results, providing a more realistic environment for studying electrochemical interactions. The simulations were performed in the canonical NVT ensemble, which keeps the number of particles, volume, and temperature constant. This allows precise control of thermodynamic variables and ensures system stability over the simulation time, enabling a detailed analysis of the system’s properties under different conditions. The temperature was set to 500 K and maintained using the velocity-rescaling (v-rescale) thermostat,^34^ with a coupling time constant of 0.1 ps. The simulation protocol was divided into two main stages: the first (∼15 ns) aimed to achieve thermodynamic equilibration, while the second (50 ns) was dedicated to the production run and subsequent property analysis. The time step used was 0.001 ps. Electrostatic interactions were treated using the Particle Mesh Ewald (PME) method,^35^ with a cutoff radius of 1.3 nm. van der Waals interactions were handled using the cutoff method,^36^ also with a 1.3 nm cutoff. Simulations were performed with Gromacs,^37^ using the LINCS algorithm^38^ to constrain bond lengths. Trajectory visualization and analysis were performed using VMD.^39^ Water molecules were described using the TIP3P model,^40^ and all graphene layer (positive and negative electrodes) and ions of the ionic liquids were modeled with the OPLS-AA force field.^41^

The electric potential across the planar electrodes was described based on the one-dimensional Poisson equation:^18,30^ $\Phi \left(z\right)=-\frac{1}{\epsilon }\int_{-z_{0}}^{z}(z-z^{\prime })\rho_{z}\left(z^{\prime }\right)dz^{\prime }$ , where Φ­(*z*) is the electrostatic potential profile along the *z*-axis and ρ_*z*_(*z*′) is the local charge density. The potential difference between the electrodes was calculated as δδΦ = δΦ_+_ – δΦ_–_, where δΦ _±_ = Φ_±_^charged^ – Φ_±_^discharged^. In molecular simulations of SCs, electrode polarization is crucial in SC simulations to accurately model EDL formation. Two main approaches exist: (i) the constant charge method (CCM) and (ii) the constant potential method (CPM).^42^ While CPM captures potential fluctuations more accurately, CCM is widely adopted due to its lower computational cost and has proven sufficient for low-voltage applications.^42^ In this study, we adopted the CCM approach, which has shown excellent agreement with experimental data in previous works.^18,30,43,44^

Based on the obtained potential profiles, a linear fitting of Φ _±_ × σ _±_ was used to calculate the capacitance of the positive and negative electrodes, *C*_+_ and *C*_–_, respectively, where the slope of the linear fit represents the capacitance. Considering the electrodes are connected in series, the total device capacitance was calculated as $C_{\mathrm{tot}}=\frac{C_{+}C_{-}}{C_{+}+C_{-}}$ . Four surface charge densities were evaluated: σ = ± 0.00 e/nm^2^ (discharged state, PZC), ± 0.10 e/nm^2^, ± 0.20 e/nm^2^, and ± 0.30 e/nm^2^. These surface charge densities was distributed over the surface area of each electrode (containing 540 carbon atoms), resulting in an atomic charge per carbon atom of the electrode of ± 0.00000000e, ± 0.00264566e, ± 0.00529132e, and ± 0.00793697e, respectively, thus representing the progressive charging process of the SCs. Finally, the gravimetric energy density of each device was estimated using $u_{m}=\frac{1}{2m}C_{\mathrm{tot}}\delta \delta \Phi^{2}$ allowing us to assess the energy storage capacity per unit mass.

## 3 Results and Discussion

### 3.1 Coulomb and Lennard-Jones Interaction Energies

First, the interaction energies between each electrolyte species and the electrodes were calculated to evaluate how the EDL is structured based on the energetic interactions between the system components. Coulomb interaction energies are shown in Figure 2, whereas Lennard-Jones (LJ) interaction energies are presented in Figure 3. For systems with electrodes subjected to a surface charge density of σ = ± 0.30 e/nm^2^, the Coulomb interactions between [bmim] cations and the positively charged graphene electrode were between 0.3 and 0.6 kcal/mol per ion. These values indicate repulsive interactions, as expected for species with the same charge. Conversely, the interactions between [bmim] and the negatively charged graphene were significantly more attractive, with values between −0.9 and −0.8 kcal/mol per ion. These results demonstrate a much stronger electrostatic affinity between [bmim] and the oppositely charged electrode, confirming the dominance of attractive forces in these configurations.

**Figure 2:**
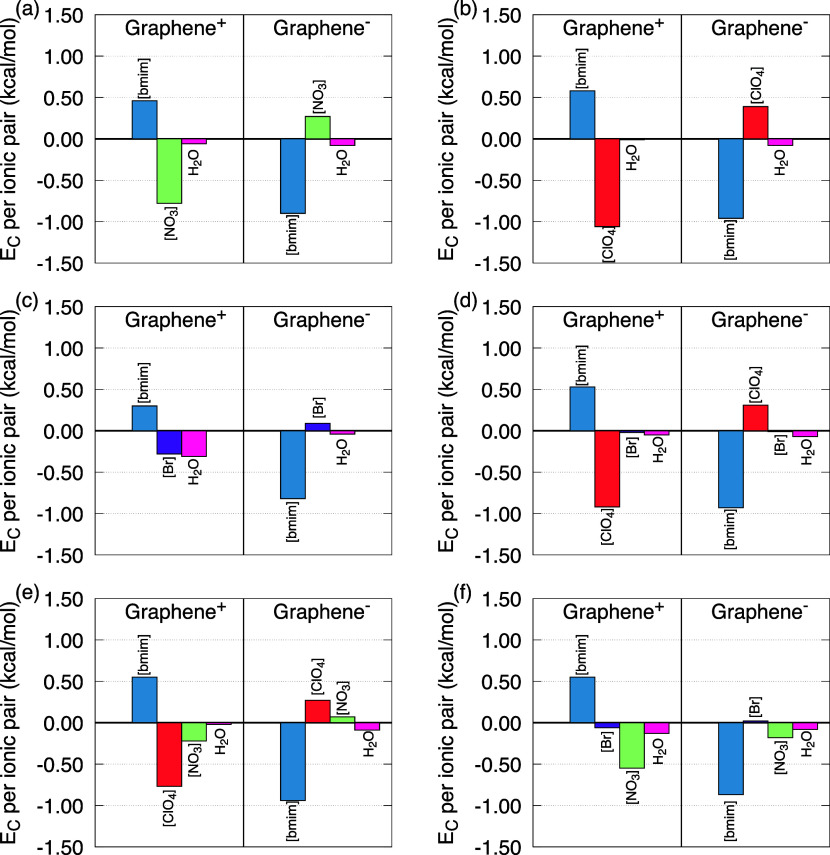
Coulomb interaction energy (*E*_C_), per ion pair, in the different models studied. The interactions between all species and the positive and negative electrodes are highlighted in the following order: (a) M1; (b) M2; (c) M3; (d) M4; (e) M5; (f) M6. The corresponding color code is shown in the image.

**Figure 3:**
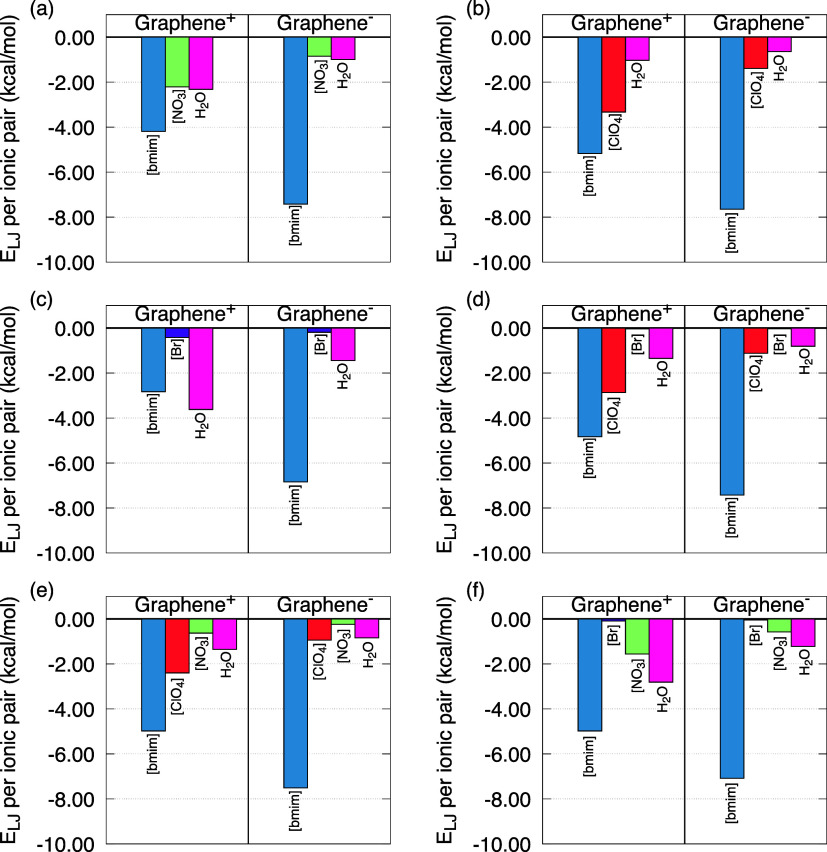
Lennard-Jones interaction energy (*E*_LJ_), per ion pair, in the different models studied. The interactions between all species and the positive and negative electrodes are highlighted in the following order: (a) M1; (b) M2; (c) M3; (d) M4; (e) M5; (f) M6. The corresponding color code is shown in the image.

In systems where the electrolyte consists of only one hydrated ionic liquid, Coulomb interactions energy (*E*_C_) between anions and the positively charged electrode were found to be attractive as expected and *E*_C_(M3) > *E*_C_(M1) > *E*_C_(M2). It is noteworthy that the anions capable of forming hydrogen bonds (HB) with water molecules, such as NO_3_^–^ and ClO_4_^–^, exhibited stronger interactions than Br^–^. This indicates that HBs significantly facilitates the proximity and adsorption of anions at the electrode surface, thus influencing the EDL structure. This trend is also observed in systems with mixed anions. For instance, in model M4, the *E*_C_(Br^–^ and Graphene^+^) > *E*_C_(ClO_4_^–^ and Graphene^+^); In model M5, *E*_C_(NO_3_^–^ and Graphene^+^) > *E*_C_(ClO_4_^–^ and Graphene^+^); and in model M6, *E*_C_(Br^–^ and Graphene^+^) > *E*_C_(NO_3_^–^ and Graphene^+^). These results reinforce the observation that HB-forming anions tend to exhibit stronger electrostatic interactions with the graphene electrodes.

With respect to anion interactions with the negatively charged electrode, repulsive Coulomb interactions were observed, as expected. The values obtained were between 0.01 and 0.40 kcal/mol per ion. The results indicate that Br^–^ plays a very limited electrostatic role in the EDL formation, consistent with its low hydrogen-bonding ability. Coulomb interactions between water molecules and the electrodes were generally weak (<−0.1 kcal/mol per ion pair). However, in systems containing Br^–^, these interactions became more pronounced: for H_2_O–Graphene^+^, values of −0.31 kcal/mol in M3 and −0.13 kcal/mol in M6 were observed. This increase may result from water compensating for the low interaction of Br^–^ with the electrode, assuming a more prominent role in stabilizing the EDL.

When analyzing Lennard-Jones interactions energy (*E*_LJ_), they were found to be dominant in magnitude and, therefore, played a central role in structuring the EDL, for this reason, we will quantitatively emphasize this property on a per ion pair basis. In model M1, for example, the *E*_LJ_ between [bmim] and the electrodes were −4.19 kcal/mol (Graphene^+^) and −7.42 kcal/mol (Graphene^–^), revealing strong attraction, particularly toward the negatively charged electrode. For NO_3_^–^, greater affinity with the positive electrode was observed with a difference of approximately 61% (−2.21 kcal/mol vs −0.85 kcal/mol). Water molecules also showed attractive interactions of −1.98 [−1.12] kcal/mol with the positive [negative] electrode. Similar behavior was observed for models M2 and M3, with [bmim] interacting via LJ potentials with −5.17 and −7.65 [−2.83 and −6.84] kcal/mol in M2 [M3] for the Graphene^+^ and Graphene^–^, respectively. In all cases, [bmim] exhibited a stronger affinity for the negatively charged electrode. For the anions, the data also suggest a preference for the electrode with the opposite charge. In M2, the *E*_LJ_(ClO_4_^–^ and Graphene) were −3.33 and −1.39 kcal/mol, respectively for positive and negative electrodes. In M3, the *E*_LJ_(Br^–^ and Graphenes) are −0.42 and −0.19 kcal/mol, reinforcing that Br^–^ weakly interacts with both electrodes. In mixed-electrolyte models (M4–M6), the strongest *E*_LJ_ values for [bmim] were observed: −4.83/–7.42 kcal/mol (M4), −4.98/–7.51 kcal/mol (M5), and −3.61/–7.08 kcal/mol (M6), again showing a preference for the negative electrode. For the anions, ClO_4_^–^ consistently exhibited stronger interactions than Br^–^. In M4, the interactions with the positive electrode were −2.87 (ClO_4_^–^) and −0.04 (Br^–^), and with the negative electrode, −1.12 and −0.03 kcal/mol, respectively. In M6, Br^–^–electrode interactions remained negligible: −0.09 kcal/mol (Graphene^+^) and −0.05 kcal/mol (Graphene^–^). Water molecules demonstrated stronger *E*_LJ_ with the positive electrode in the single-RTIL models: −2.32 (M1), −1.39 (M2), and −3.62 kcal/mol (M3) for Graphene^+^, compared to −0.99, −0.64, and −1.45 kcal/mol for Graphene^–^. The presence of Br^–^, especially in M3, appears to enhance water–electrode interactions, suggesting that in systems with less interactive ions, water molecules take on a crucial role in shaping the EDL.

The energetic analysis presented in Table 2 illustrates the behavior of the total interaction energies (in kJ/mol per ion pair or per water molecule) among the main components of the studied electrolyte systems: [bmim] cations, anions, and water molecules. For the traditional systems containing a single anion (M1, M2, and M3), a significant variation is observed in the water–anion interactions. The system with bromide (M3) exhibits the strongest water–anion interaction (−210.42 kJ/mol), followed by the nitrate system (M1) with an intermediate interaction (−173.74 kJ/mol), and the perchlorate system (M2), which shows the weakest water–anion interaction (−120.02 kJ/mol). These differences reflect the distinct hydration behavior of each anion, associated with their varying abilities to form hydrogen bonds and electrostatic interactions with water molecules. The cation–water interactions are consistently strong across all systems, indicating favorable solvation of the [bmim] cations by water molecules. Among them, the system with bromide (M3) shows the most intense cation–water interaction (−75.28 kJ/mol), suggesting greater stabilization of the cations in this medium. In the mixed systems (M4, M5, and M6), the interaction energies reflect a combination of the effects observed in the single-anion systems. For example, system M4 (containing ClO_4_^–^ and Br^–^) exhibits moderate water–anion interactions for both anions (−52.73 and −109.21 kJ/mol, respectively), while systems M5 (ClO_4_^–^ and NO_3_^–^) and M6 (Br^–^ and NO_3_^–^) show a balance between the energetic interactions of their constituent ions with water molecules. These mixed systems also tend to maintain favorable hydration characteristics. Finally, water–water interactions are consistently strong across all systems, reflecting the hydrogen-bonding network intrinsic to the solvent. Small variations in these values indicate the influence of ionic species on water structuring within the electrolyte. Overall, these values highlight the critical role of specific ion interactions in shaping the microenvironment of hydrated RTIL-based electrolytes.

**Table 2:** Total Interaction Energy (Coulomb + Lennard-Jones), in kJ/mol Per Number of Particles, for Each Studied System^a^

electrolyte	[bmim] – H_2_O	[NO_3_] – H_2_O	[ClO_4_] – H_2_O	[Br] – H_2_O	H_2_O – H_2_O
M1	–65.78	–173.74			–823.11
M2	–55.05		–120.02		–825.31
M3	–75.28			–210.42	–768.28
M4	–65.01		–52.73	–109.21	–804.35
M5	–60.41	–89.70	–56.75		–827.45
M6	–70.38	–83.07		–83.07	–789.08

a Results obtained for the charged SC.

### 3.2 Ionic Species vs Graphene Electrodes Total Interaction Energy

To better understand which ionic species, dominate interactions with the graphene electrodes, we separately analyzed the interaction energy data for the systems containing mixed electrolytes (models M4 to M6) under the condition of highest surface polarization (σ = ± 0.30 e/nm^2^). It is important to note that, in all analyzed electrolytes, the molar ratio between [bmim] and the anions is 2:1—meaning there are twice as many cations as anions. Therefore, to ensure a fair comparison of individual contributions, the total interaction energy attributed to the cation was divided by two. In system M4, the analysis shows that after stoichiometric adjustment, the ClO_4_^–^ anion dominates the interactions with the positive electrode, exhibiting a more negative interaction energy (*E*_C_ + *E*_LJ_ = – 2.56 kcal/mol) than the adjusted [bmim] cation (*E*_C_ + *E*_LJ_ = −2.15 kcal/mol) and other components. On the negative electrode, however, the cation remains the dominant species, even after adjustment (*E*_C_ + *E*_LJ_ = −3.18 kcal/mol). For the M5 model, the adjusted contributions indicate that both [bmim] and ClO_4_^–^ interact similarly with the positive electrode (*E*_C_ + *E*_LJ_ = −2.22 and −2.13 kcal/mol, respectively), suggesting a cooperative behavior at the positively charged graphene interface. Conversely, at the negative electrode, the dominance of the [bmim] cation remains clear (*E*_C_ + *E*_LJ_ = −4.14 kcal/mol), and considerably greater than those of the anions. In the M6 electrolyte, a distinct behavior is observed. The NO_3_^–^ anion clearly dominates the interactions with the positive electrode (*E*_C_ + *E*_LJ_ = −2.11 kcal/mol), surpassing the adjusted contribution of [bmim] (*E*_C_ + *E*_LJ_ = −1.61 kcal/mol). This may be related to its high charge density and structural symmetry on EDL interface. Nevertheless, at the negative electrode, [bmim] remains the predominant species (*E*_C_ + *E*_LJ_ = – 3.57 kcal/mol), still more negative than those of the anions and water molecules. Overall, even when accounting for the higher proportion of [bmim]^+^, the results indicate that the cation consistently plays the main role in interactions with the negatively charged electrode. On the other hand, dominance at the positively charged electrode varies depending on the nature of the anion, with ClO_4_^–^ and NO_3_^–^ being the main contenders (all calculated interaction energies are provided in the Supporting Information – Tables S1–S4).

### 3.3 Number Density Analysis

Based on the energetic analysis of *E*_C_ and *E*_LJ_, we computed the normalized number density (NND) to the bulk values for each system to investigate the spatial distribution of molecules/ions near the positively and negatively charged electrodes. This behavior is depicted in Figure 4. Overall, the trends observed in the energetic analysis are clearly reflected in the density profiles. For instance, in model M1 (Figure 4a), there is evident spatial competition between [bmim] and H_2_O molecules in the vicinity of the positively charged electrode. This competition is reflected by comparable density peaks for these two species, while the NO_3_^–^ anions display a much smaller peak, indicating reduced adsorption. The significant presence of water molecules near the electrode suggests enhanced dilution of the EDL and highlights the importance of HB networks (between water molecules and with other species) in shaping the interfacial structure.

**Figure 4:**
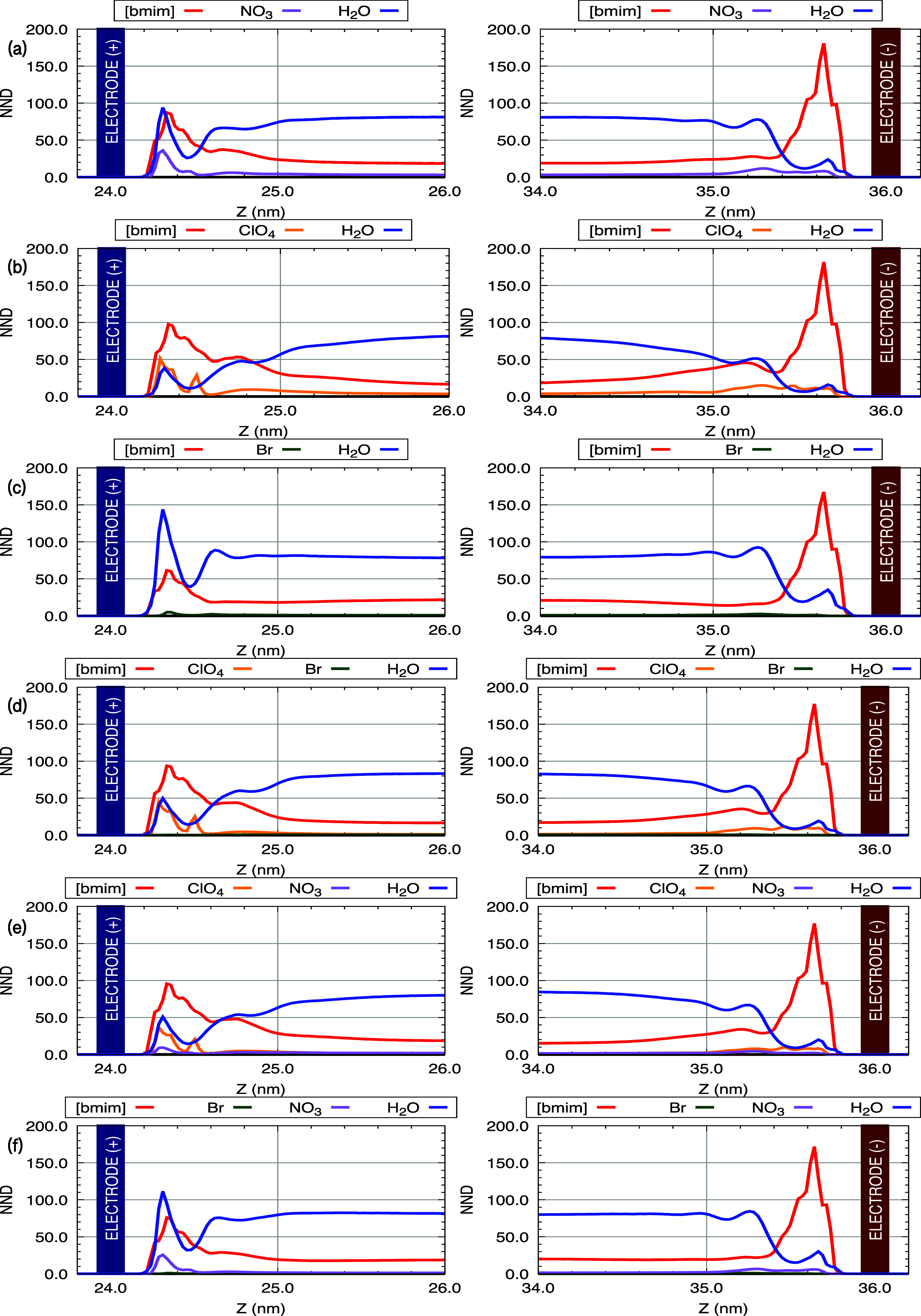
Normalized number density (NND, in number of molecules or ions per nm^3^) profiles with respect to the bulk along the *Z*-axis for the models: (a) M1; (b) M2; (c) M3; (d) M4; (e) M5; (f) M6.

A similar behavior is observed in models containing anions capable of forming HBs, such as ClO_4_^–^ and NO_3_^–^ (M2, M4, M5, and M6). In these cases, water molecules are found in large amounts near the positively charged electrode, while the anions show moderate density peaks, competing for space at the graphene’s interface. Still, the highest density values consistently correspond to [bmim], indicating its strong adsorption, in agreement with the interaction energy results. In contrast, systems containing Br^–^ anions (M3, M4, and M6) reveal that this species barely reaches the interfacial region. In M3 (Figure 4c), for example, the NND­(Br^–^) is nearly zero throughout the positive graphene, while water molecules show the highest peak, followed by [bmim]. This lack of Br^–^ accumulation reinforces its weak interaction with the electrode and its inability to form HBs, reducing its contribution to the electrode–electrolyte structuring. On the negative electrode side (right panels), the trend is reversed. All models show a pronounced peak of [bmim] near the negatively charged electrode, indicating strong adsorption—consistent with the attractive interaction energies observed for this pair. A second peak, generally associated with water molecules, is typically located further from the electrode surface, suggesting the formation of a hydrated layer. Meanwhile, the anions generally exhibit very low densities in this region, which aligns with the repulsive E_C_ identified in the energetic analysis. In summary, the NND profiles confirm that the EDL structure is strongly influenced by the specific affinity of each species toward the electrodes, as well as by their ability to engage in HB.

### 3.4 Hydrogen Bonds Analysis

As previously demonstrated, the presence of species capable of forming HBs significantly alters the structure of the EDL, and consequently, the electrochemical performance of the devices. To investigate this phenomenon in more detail, we selected 10^4^ configurations from the MD trajectory for the systems with a surface charge density σ = ± 0.30 e/nm^2^ and divided each one into three distinct regions: EDL^+^, EDL^–^ (up to 2 nm from the electrode), and the bulk (central region of SC, *d* = 8 nm). The objective was to quantitatively evaluate the average number of HBs in each region, as well as to count the average abundance of each species across the different domains of each system. The HBs analysis (per water molecule) in [bmim]-based RTIL models with water molecules reveals how the nature of the anion directly affects the structuring of water within the EDL^+^, bulk, and EDL^–^ regions. Two main types of interactions were considered: water–water and anion–water.

In Figure 5a (model M1), a strong interaction between NO_3_^–^ and water molecules is observed. The average number of HBs formed by each water molecule in the EDL^+^ and EDL^–^ regions is approximately 0.30, whereas in the central region of the supercapacitor, this value does not exceed 0.20. This suggests an accumulation of NO_3_^–^ near the electrodes and high coordination ability via HBs with water molecules. It is also noteworthy that, despite these regional differences, the HB network between water molecules (water–water) shows little variation across the three regions of the system, with average values ranging from approximately 1.20 to 1.30 HBs per water molecule. Model M2 (Figure 5b) presents a different scenario. The HB between ClO_4_^–^ and water molecules contributions are lower (<0.20 in EDLs and <0.10 in the bulk), allowing for better preservation of the H_2_O–H_2_O structure, which remains between 1.10 and 1.30 in the EDLs and bulk of SC. This indicates that the less coordinating nature of ClO_4_^–^ favors structural stability of the water network even near the electrodes. In model M3 (Figure 5c) the profile is even more distinct. Since Br^–^ does not form HBs with water molecules, Br^–^–water interactions are not observed. All detected HBs are of the water–water type, with averages ∼1.14 in the EDLs regions, increasing slightly to ∼1.22 in the bulk. This finding confirms that the presence of the Br^–^ ion does not significantly affect the hydrogen-bonding network among water molecules. In model M4 (Figure 5d), it is observed that the mixture containing ClO_4_^–^ and Br^–^ slightly influences the average number of hydrogen bonds formed between the ClO_4_^–^ ion and water molecules. However, the water–water hydrogen-bonding structure remains like that of the previous models. This suggests that in the absence of strongly coordinating anions, the water network remains preserved even in interfacial regions, potentially contributing to electrolyte stability. In the M5 model, the number of HBs between NO_3_^–^–water and ClO_4_^–^-water in the EDL regions is below 0.20 per water molecule. In the M6 model, NO_3_^–^–water interactions remain around 0.14, with no significant contribution from Br^–^–water interactions. In both models, no changes are observed in the water–water hydrogen-bonding behavior when compared to the other systems. These analyses suggest that ion–water interactions in the individual RTILs (Models M1, M2, and M3) tend to be slightly more intense than those in the mixed systems (Models M4, M5, and M6).

**Figure 5:**
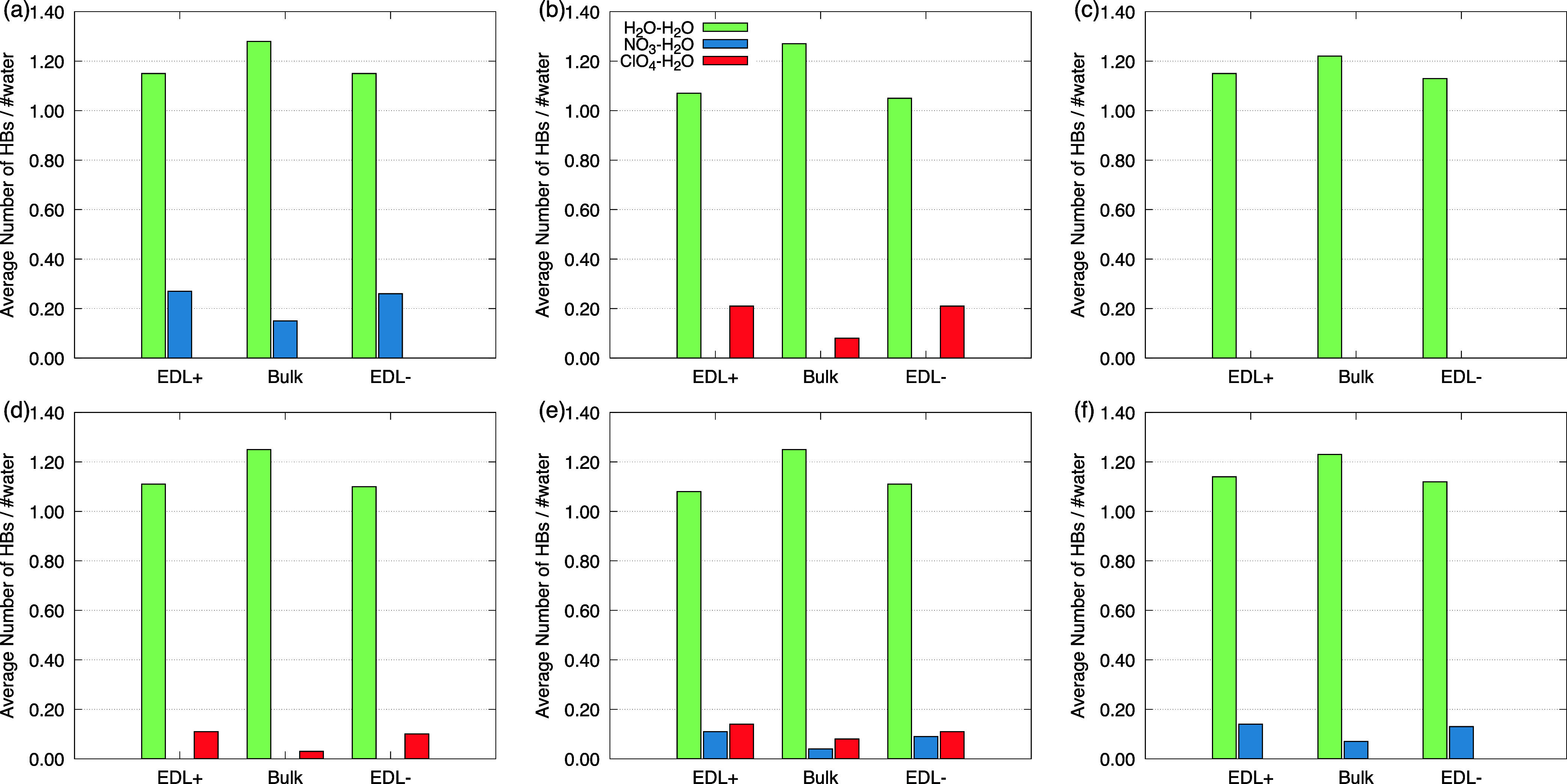
Average number of hydrogen bonds (per water molecule) for the studied models. The panels follow the order: (a) M1; (b) M2; (c) M3; (d) M4; (e) M5; (f) M6. Green = Water–Water interactions; Blue = NO_3_^–^– water interactions; and red ClO_4_^–^– water interactions.

### 3.5 Species Distribution Across EDLs and Bulk Regions of the SCs

The structural organization of electrolytes based on [bmim]-type ionic liquids with different anions in aqueous media reveals distinct spatial distribution patterns for the species in the EDL regions (EDL^+^ and EDL^–^) and the central bulk region of the system, additionally, the HBs statistics reveal a slight influence of the ions within the regions of interest, yet this does not significantly affect the structure of water–water interactions. To gain deeper insights into these behaviors, we quantitatively analyzed the average number of cations, anions, and water molecules in each region (see Table 3), allowing us to identify clear trends of ion segregation, accumulation, and solvation that define the local microstructure of each system.

**Table 3:** Distribution of the Species Present in the Electrolytes for All Analyzed Models^a^

electrolyte	species	EDL^+^	Bulk	EDL^‑^	Q EDL^+^	Q bulk	Q EDL^‑^
traditional-RTILs systems (2 M in water solution)							
M1	[bmim]	30	85	37	–0.17	0.00	+0.21
	NO_3_^–^	35	85	31			
	water	584	3098	538			
M2	[bmim]	38	67	43	–0.17	–0.01	+0.21
	ClO_4_^–^	43	68	37			
	water	468	3200	438			
M3	[bmim]	23	97	32	–0.21	0.00	+0.17
	Br^–^	29	97	27			
	water	657	3002	587			
mixed-RTILs systems (2 M in water solution)							
M4	[bmim]	33	79	38	–0.17	–0.01	+0.21
	ClO_4_^–^	27	27	21			
	Br^–^	11	53	11			
	water	536	3139	509			
M5	[bmim]	36	75	37	–0.17	0.00	+0.21
	ClO_4_^–^	25	30	18			
	NO_3_^–^	16	45	13			
	water	501	3155	518			
M6	[bmim]	26	92	34	–0.21	+0.01	+0.21
	Br^–^	12	52	12			
	NO_3_^–^	20	39	17			
	water	627	3048	564			

a This analysis considered 10^4^ configurations selected from the molecular dynamics (MD) trajectory, with the regions corresponding to the electric double layers (EDLs) defined as 2 nm from the electrodes. *Q* represents the total net charge density (for ions species) in the EDL and bulk regions, normalized by EDL or Bulk’s volume (in units of e/nm^3^). Volume of ELD = 28.57 nm^3^ and volume of bulk = 114.30 nm^3^.

In model M1, a relatively symmetric distribution of [bmim] and NO_3_^–^ ions is observed near the electrodes, with approximately 65 and 68 ions in the EDL^+^ and EDL^–^ regions, respectively. However, this symmetry does not extend to water molecules, which are distributed as 584 in EDL^+^ and 538 in EDL^–^ — representing a reduction of about 8%. In model M2, the ion distribution is also symmetric, with an average of 81 and 80 ions in the EDL^+^ and EDL^–^ regions. Nonetheless, a slight preferential accumulation of cations near the negative electrode and anions near the positive electrode is observed. Again, a reduction in water content is found in EDL^–^, amounting to approximately 7% less than in EDL^+^. Model M3 exhibits one of the most distinctive distributions. The number of ions at the interfaces is lower — about 52 and 59 in the EDL^+^ and EDL^–^, respectively — while water molecules are more prevalent, with approximately 657 in EDL^+^ and 587 in EDL^–^. This corresponds to a systematic reduction of about 12% in water content near the negative electrode. For these three models composed of pure RTILs, it is also notable that 70–78% of water molecules are in the bulk region of the supercapacitor. Regarding ion distribution in the bulk, model M2 shows the lowest proportion, with only about 45% of the ions located in this region, while models M1 and M3 indicate approximately 56 and 64%, respectively. Despite these differences, the net charge distribution of the RTILs near the electrodes remains relatively balanced, with values around −5e at the positive electrode and +6e at the negative electrode. In terms of volumetric charge density, these values correspond to approximately −0.17 and +0.21 e/nm^3^ (considering a volume of 28.58 nm^3^ in the EDL regions).

In mixed-anion systems, more complex patterns emerge. In M4 model, ClO_4_^–^ is evenly distributed (∼27 in the bulk and ∼22–27 in the EDLs), while Br^–^ concentrates strongly in the bulk (∼53) and is less abundant at the interfaces (∼11). Water is evenly distributed, with over 500 molecules in each EDL and ∼3139 in the bulk. This combination of a weakly structuring anion (ClO_4_^–^) and a noninteracting one (Br^–^) leads to well-hydrated interfaces. In model M5 the distribution becomes more asymmetric. Although NO_3_^–^ [ClO_4_^–^]­appears in lower absolute HB numbers in the EDLs (∼13 and ∼16 [∼25 and ∼18]), its strong affinity for HB. The bulk contains the highest concentration of both species (∼30 and ∼45), along with over 3150 H_2_O molecules. Finally, model M6 combines two extremes: a highly hydrated anion (NO_3_^–^) and a nonstructuring one (Br^–^). NO_3_^–^ is strongly localized in the EDLs regions (∼19 in EDL^+^ and ∼17 in EDL^–^) which corresponds to approximately 9.5 and 8.5 ions per unit length in this region (2 nm), while in the bulk (∼39), there are about 4.9 ions of this species per unit length (8 nm). In contrast, Br^–^ exhibits a more uniform distribution, with around 6.0–6.5 ions per nm throughout the interior of the SC. Water is abundant throughout the system, with peaks of 627 in EDL^+^ and 564 in EDL^–^. Despite its lower relative concentration, NO_3_^–^ continues to influence the graphene-electrolyte interface. These results demonstrate that both the local concentration and chemical nature of species within each region strongly influence electrolyte structuring. Strongly coordinating anions like NO_3_^–^ tend to accumulate at the interfaces, while weakly coordinating ones like ClO_4_^–^ and Br^–^ coexist with a more extensive network of HBs, particularly in the bulk. Thus, electrolyte composition can be strategically modeled to modulate structural organization of EDLs and/or electrochemical performance in hydrated ionic liquid-based supercapacitors.

The findings underscore the robustness of the adopted methodology and demonstrate that EDLs formation is a highly cooperative process, in which charge balance takes precedence over the individual affinity of ionics species for specific regions. Furthermore, they suggest that the local EDL structure is not solely governed by the chemical nature of the ions but also by the requirement to neutralize the electrode surface charge, which can drive complex. This point is fundamental to understanding the behavior of hydrated ionic liquid-based SCs, as it reveals that charge storage capacity does not rely solely on specific ions–electrodes interactions but also on the EDLs’ ability to organize.

### 3.6 Electrochemical Performance Analysis

The resulting values of the electrostatic potentials (δδϕ) are presented in Table 4. Under high surface polarization conditions, represented by a charge density of σ = 0.30 e/nm^2^, all analyzed electrolytes exhibited a clear response in terms of developing an electric potential difference between the electrodes, reflecting the efficiency of EDL formation. The total potential difference, calculated as δδϕ = δϕ_+_ – δϕ_–_, varied slightly across systems, ranging from 1.98 to 2.11 V. Although the variation lies within a relatively narrow range, these differences are significant from the perspective of electrolyte structural organization and reflect the specific properties of each ionic composition. The systems that showed the highest δδϕ values were M2 and M3, both reaching a total potential difference of 2.11 V. The combination with less coordinating anions — such as ClO_4_^–^ in M2 and Br^–^ in M3 — appears to favor efficient charge separation without hindering the formation of EDLs, contributing more effectively to the increase of the device’s electrochemical window. System M4 presented a δδϕ = 2.08 V, close to the values observed in the previous cases. This suggests that even in the absence of NO_3_^–^, a strong electrostatic response can still be achieved, provided there is a suitable balance between ionic and water organization. Conversely, when only ClO_4_^–^ and Br^–^ are present, as in system M5, the δδϕ value was among the lowest observed, reaching 1.98 V, slightly higher than the 1.97 V recorded for M6. This may be attributed to the weak interaction of these anions with water molecules and the electrodes, resulting in a less efficient EDL.

**Table 4:** Description of the Electrode Potentials Obtained^a^

electrolyte	σ (e/nm^2^)	Φ_+_ (V)	Φ_–_ (V)	δΦ (V)	δδΦ (V)
traditional-RTILs systems					
M1	0.00	0.69	0.74	–0.04	–0.04
	0.10	1.00	0.33	0.66	0.71
	0.20	1.28	–0.07	1.35	1.39
	0.30	1.52	–0.47	1.99	2.04
M2	0.00	0.60	0.62	–0.03	–0.03
	0.10	0.91	0.18	0.73	0.75
	0.20	1.17	–0.17	1.34	1.37
	0.30	1.50	–0.58	2.08	2.11
M3	0.00	0.85	0.86	–0.03	–0.03
	0.10	1.10	0.49	0.73	0.75
	0.20	1.35	0.09	1.73	1.75
	0.30	1.56	–0.31	2.08	2.11
mixed-RTILs systems					
M4	0.00	0.66	0.68	–0.02	–0.02
	0.10	0.95	0.30	0.65	0.67
	0.20	1.27	–0.12	1.39	1.41
	0.30	1.56	–0.49	2.06	2.08
M5	0.00	0.65	0.65	0.00	0.00
	0.10	0.93	0.28	0.64	0.65
	0.20	1.23	–0.14	1.37	1.37
	0.30	1.48	–0.50	1.98	1.98
M6	0.00	0.77	0.76	0.02	0.02
	0.10	1.04	0.38	0.66	0.64
	0.20	1.29	0.01	1.29	1.27
	0.30	1.56	–0.43	1.99	1.97

a Highlighted values include the potential at the positive electrode (ϕ_+_), the negative electrode (ϕ_–_), the potential difference (δϕ), and the total potential difference corrected by the point of zero charge (PZC, σ = 0.00 e/nm^2^), denoted as δδϕ, for all analyzed surface charge densities.

The M1 model, composed solely of the hydrated RTIL [bmim]­[NO_3_^–^], exhibits an electric potential difference of approximately 2.04 V—about 0.08 V (∼4%) higher than that of the M6 model, which involves an RTIL mixture containing Br^–^ ions and a reduced concentration of NO_3_^–^ ions. This change does not alter the charge distribution in the EDL, as previously observed, but it does affect the resulting electric potential due to the distinct ways these ions interact with water molecules and the electrodes, as highlighted earlier. This emphasizes a more intricate context in the dynamics of the species forming the EDLs, particularly when these species can form hydrogen bonds.

### 3.7 Supercapacitors’ Capacitance

Based on the potential values, a linear fit of the form *f*(*x*) = *ax* + *b* was performed for the relationship σ _±_ × ϕ _±_, where the slope of the fitted lines corresponds to the capacitance of the positive and negative electrodes, see Figure 6. The capacitance values (in μF/cm^2^) for each electrode (*C* ^+^ and *C* ^–^), as well as the total capacitance (*C* ^Tot^) of the device, are presented in Table 5. The *C* ^+^ ranged from 5.27 to 6.78 μF/cm^2^, while those for *C* ^–^ were between 3.97 and 4.14 μF/cm^2^. Considering that the electrodes are connected in series, the *C* ^Tot^ range between 2.30 and 2.55 μF/cm^2^. Among all the systems analyzed, the highest total capacitance was observed for model M3, corresponding to the [bmim]­[Br] electrolyte, which reached 2.55 μF/cm^2^. This result is particularly noteworthy because, as shown in our structural analyses, model M3 presents the lowest ion density within the EDL regions, and no HBs are observed between ions and water molecules. Despite the low density of structuring species at the interfaces, the net charge within the EDLs remains constant due to the fixed-charge condition imposed on the electrodes during the MD simulation. One possible explanation for the good capacitive performance of M3 is the small size of the Br^–^ ion and its accessibility to the electrode surface, promoting adsorption and improved EDLs organization, which directly contributes to increased *C* ^Tot^.

**Figure 6:**
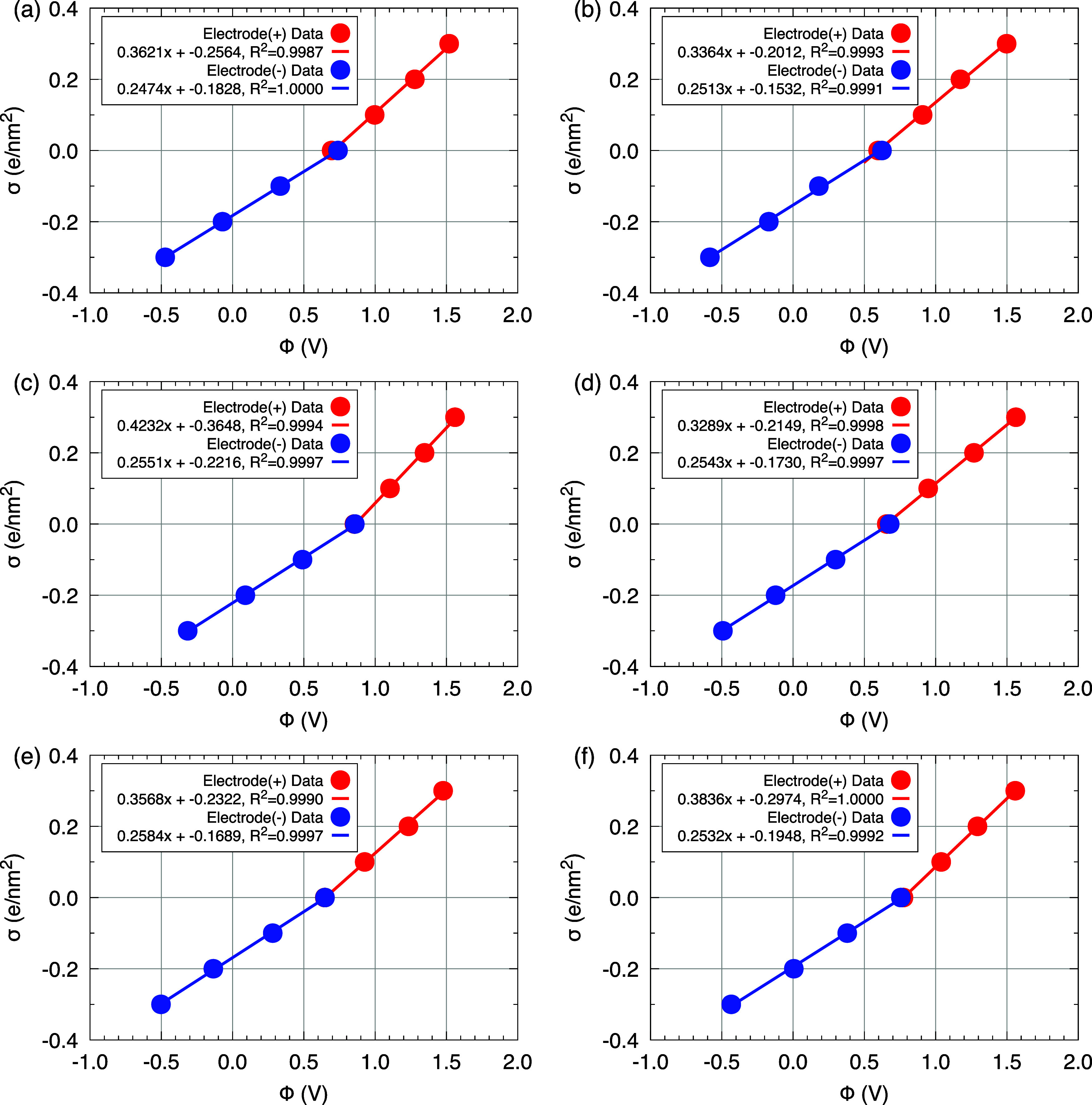
Graphical representation of σ × ϕ for the studied models (a–f). The linear fit is given by *f*(*x*) = *ax* + *b*, where the slope of the line represents the electrode capacitance. The Pearson correlation coefficients are also highlighted.

**Table 5:** Obtained Values for the Electrode Capacitances (in μF/cm^2^)­^a^

electrolyte	*C* ^*+*^	*C* ^*–*^	*C* ^Tot^
traditional-RTILs systems			
M1	5.80	3.97	2.36
M2	5.39	4.03	2.31
M3	6.78	4.09	2.55
mixed-RTILs systems			
M4	5.27	4.08	2.30
M5	5.72	4.14	2.40
M6	6.15	4.06	2.45

a The partial (*C* ^+^ and *C* ^–^) and total capacitance (*C* ^Tot^) of the device, calculated using the series capacitor relationship, is also highlighted.

In systems containing single-ion electrolytes (such as M1 and M2), the *C* ^Tot^ values were more modest, ranging from 2.31 to 2.36 μF/cm^2^. These outcomes reflect the structural characteristics of the respective anions. For systems composed of RTIL mixtures (M4, M5, and M6), the *C* ^Tot^ values ranged from 2.30 to 2.45 μF/cm^2^. The lowest value was recorded for model M4, which combines [bmim]­[Br] and [bmim]­[*ClO*_4_] RTILs — two anions with poor affinity for water. In contrast, models M5 and M6 showed slightly higher values in mixtures models, reaching 2.40 and 2.45 μF/cm^2^, respectively. This improvement can be attributed to the presence of NO_3_^–^, whose high affinity for water.

Our results demonstrate that the systems investigated in this study exhibit strong application potential, particularly when compared to data from previous works.^30,44^ For instance, Chagas et al.^44^ evaluated devices based on graphene electrodes and the ionic liquid [emim]­[BF_4_], reporting specific capacitances of 2.48 and 2.55 μF/cm^2^—values that are within the same range as those obtained in the present study. In other cases, our models outperformed previous systems. When compared with studies in ref [^30^] which examined graphene-based devices using [bmim]­[PF_6_] (1.89 μF/cm^2^) and a 2 M mixture of [cho]­[gly]:[bmim]­[PF_6_] (2.03 μF/cm^2^), our model M3 — showing the highest capacitance—demonstrated superior performance, with relative increases of approximately 26 and 20%, respectively.

### 3.8 Gravimetric Energy Density Analyses

Based on the total capacitance values of the devices and the corresponding potential differences corrected by the point of zero charge (PZC), it was possible to calculate the gravimetric energy density (*u*_*m*_, in J/g) stored by each model. The results obtained for the different surface charge densities are summarized in Table 6. The data analysis reveals that, as expected, increasing the surface charge density leads to a quadratic increase in the stored energy density. For σ = 0.30 e/nm^2^, the *u*_*m*_ values ranged from 3.35 to 3.97 J/g, with the highest value observed for model M3. This system stood out with 3.97 J/g, followed closely by M2 with 3.60 J/g, and M1 with 3.52 J/g. The mixed-anion models exhibited slightly lower values, with M6 reaching 3.39 J/g, M5 at 3.35 J/g, and M4 showed the lowest value (3.28 J/g). To allow a consistent comparison, a quadratic model of the form *g*(*x*) = α*x* ^2^ was fitted for each system, correlating *u*_*m*_ with the total potential difference δδΦ. Based on these fits, it was possible to extrapolate the *u*_*m*_ values to a fixed potential difference of 2.5 V, representing an idealized operating condition. This energy projection is illustrated in Figure 7.

**Table 6:** Obtained Values for the Gravimetric Energy Density (in J/g) of the Systems Analyzed in This Study

model	σ (e/nm^2^)	*u*_*m*_ (J/g)
traditional-RTILs systems		
M1	0.10	0.42
	0.20	1.65
	0.30	3.52
M2	0.10	0.46
	0.20	1.52
	0.30	3.60
M3	0.10	0.51
	0.20	2.75
	0.30	3.97
mixed-RTILs systems		
M4	0.10	0.36
	0.20	1.61
	0.30	3.48
M5	0.10	0.36
	0.20	1.61
	0.30	3.35
M6	0.10	0.36
	0.20	1.40
	0.30	3.39

**Figure 7:**
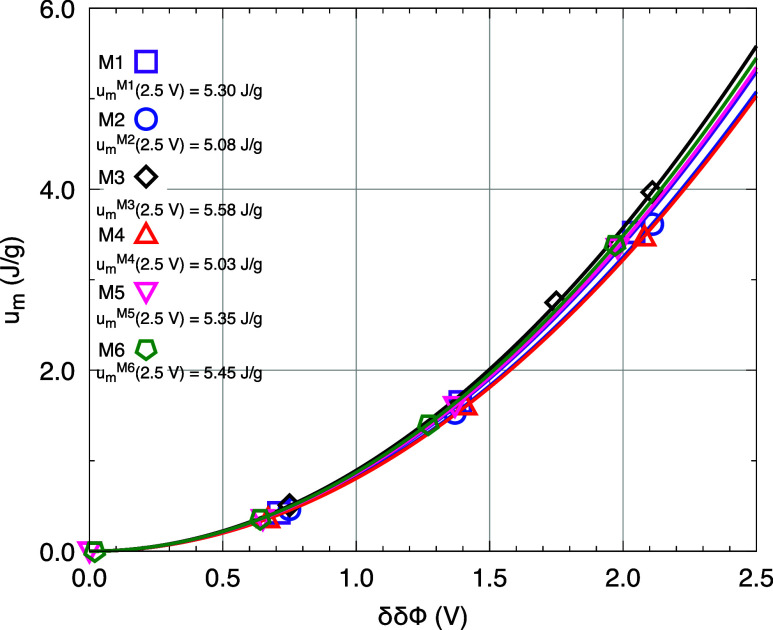
Graphical representation of u_m_ × δδϕ for the studied models. The linear fit is given by *g*(*x*) = α*x* ^2^.

Under this standardized operating condition (δδΦ = 2.5 V), model M3 continues to exhibit the best energy performance, with a projected energy density of 5.58 J/g. The second-best result was observed for model M6, reaching 5.45 J/g. This finding reinforces the structural analyses, which showed that although the Br^–^ anion contributes positively to the electrochemical performance of the devices. This effect can be attributed to the small size of the Br^–^ anion, which enhances its mobility and facilitates penetration into the EDLs regions. In contrast, the combination of Br^–^ with the ClO_4_^–^ anion (model M4) resulted in the lowest projected energy density (5.03 J/g), which is consistent with the weakly interactive structural nature of both anions. Models containing the NO_3_^–^ anion, such as M1 and M5, showed intermediate performance, reaching 5.30 and 5.35 J/g, respectively. Therefore, the projected gravimetric energy density results clearly demonstrate that the ionic composition of the electrolytes has a direct and measurable impact on the electrochemical performance of the devices. Small and mobile anions, such as Br^–^, prove to be particularly promising. Performance optimization through the careful selection of anions emerges as an effective strategy for maximizing the energy efficiency of hydrated ionic liquid-based SCs.

Indeed, certain simplifications are adopted during the modeling process to reduce computational costs without compromising the essential physical and chemical phenomena under investigation. For instance, the electrodes are typically represented with a total mass considerably smaller than that expected in real-world applications. This is because the simulations focus exclusively on the electrode–electrolyte interface, while modeling a fully realistic electrode would be computationally prohibitive. Such an approach would render comparative studies (requiring extensive and repeated simulations) practically unfeasible. However, meaningful comparisons can still be made with similar systems. A direct comparison with the models reported in ref 1,24 is appropriate, as the same surface charge density was applied to the electrode terminals. We observed that all our models exhibited slightly higher gravimetric energy densities compared to the Cl^–^ and Br^–^-based systems analyzed in those studies. Specifically, taking our M3 model at a surface charge density of 0.3 e/nm^2^ as a reference, the relative differences range from 14 to 22%. On the other hand, when comparing our models with those presented in ref [^18^] we find that they exhibit higher gravimetric energy densities than devices using pure amino acid–based electrolytes. However, under conditions of high hydration, the models proposed in this study show lower values. Specifically, for pure electrolytes such as [emim]­[ala], [emim]­[val], [emim]­[leu], and [emim]­[ile], the differences range from 1 to 21%. In contrast, for highly hydrated systems—particularly those with 90% water content—the gravimetric energy densities of our models are 22 to 36% lower, indicating that electrolyte concentration plays a significant role in determining device performance.

As previously discussed, computational modeling inherently involves certain limitations. As a result, the models employed in this work offer valuable molecular-level insights into structural and energetic trends, and quantitative comparisons with similarly constructed models are indeed meaningful. However, comparisons with experimental systems should be interpreted with caution. Real devices often exhibit more complex interfacial phenomena, diverse surface chemistries, and heterogeneous electrode morphologies that are not fully represented in idealized simulation environments. Accordingly, future studies may benefit from incorporating polarizable or reactive force fields and integrating computational results with experimental validation to more effectively bridge the gap between simulation and real-world applications.

## 4 Conclusions

The results presented in this study clearly demonstrate that the ionic composition of hydrated ionic liquids plays a decisive role in the structuring of the EDL and, consequently, in the electrochemical performance of SCs simulated. A detailed analysis of interaction energies, total potential difference (δδΦ), specific capacitance, and gravimetric energy density (*u*_*m*_) revealed consistent correlations between anion identity and both structural and functional properties of the systems. Among the evaluated electrolytes, the [bmim]­[Br] system exhibited the best overall performance. Despite showing the lowest total interaction energy, it achieved the *C* ^Tot^ = 2.55 μF/cm^2^ and the highest projected gravimetric energy density (*u*_*m*_ = 5.58 J/g). This is attributed to the small size and high mobility of the Br^–^ anion. The NO_3_^–^ anion showed intermediate performance, in the M1 model it yielded a *C* ^Tot^ = 2.36 μF/cm^2^ and projected *u*_*m*_ = 5.30 J/g, supported by its strong ability to structure the EDL through hydrogen bonding with water. In contrast, ClO_4_^–^ exhibited the strongest total interaction energy (*E*_C_ + *E*_LJ_), lower capacitance (*C* ^Tot^ = 2.31 μF/cm^2^), and projected energy storage capacity equal to 5.08 J/g.

Mixed-RTILs models demonstrated that combining complementary ionic properties can improve device electric performance. The M6 mixture reached *u*_*m*_ = 5.45 J/g, effectively combining Br^–^ mobility with NO_3_^–^ structuring capability. On the other hand, M4 model mixture exhibited the lowest projected energy density (*u*_*m*_ = 5.03 J/g), suggesting a lack of synergistic behavior between these two anions. Overall, the findings highlight that rational tuning of anion composition is a promising and effective strategy for enhancing both the EDL’s structural organization and energy performance of hydrated RTILs-based SC, paving the way for the development of more efficient and application-tailored energy storage systems.

## Supplementary Material



## Notes

Tables of Coulomb and Lennard-Jones interaction energies between ionic species and graphene electrodes for hydrated [bmim]-based ionic liquid models (M1–M6) at varying surface charge densities (PDF)
