# Supplementary material for: Evaluation of Structural and Electrochemical Properties of Supercapacitors with Graphene Electrodes and Hydrated Pure or Mixed [bmim]-Based Ionic Liquids via Molecular Dynamics
Source: ACS Phys Chem Au. 2025 Jul 15;5(5):519–32. doi: 10.1021/acsphyschemau.5c00036 (PMC12464776; doi:10.1021/acsphyschemau.5c00036)
Supplement: Supplementary file 1 [file pg5c00036_si_001.pdf]

S U P P O R T   I N F O R M A T I O N

Evaluation of Structural and Electrochemical Properties of Supercapacitors with Graphene Electrodes and Hydrated Pure or Mixed [bmim]-Based Ionic Liquids via Molecular Dynamics

Lucas de S. Silva[1] and Guilherme Colherinhas[1*]

1. Instituto de Física. Universidade Federal de Goiás. 74690-900. Goiânia. GO. Brazil.

Table S1 – Coulomb interaction energy values between all species and the electrodes (in kcal/mol) for the models composed of a single hydrated ionic liquid (models M1 to M3).

Model	Ionic Pairs	σ (e/nm^2)	[bmim] – Graphene+	[bmim] – Graphene-	[Anion] – Graphene+	[Anion] – Graphene-	H_2O – Graphene+	H_2O – Graphene-
M1	152	0.00	-	-	-	-	-	-
		0.10	0.21	-0.26	-0.21	0.15	0.00	-0.01
		0.20	0.37	-0.56	-0.48	0.24	-0.02	-0.04
		0.30	0.46	-0.90	-0.78	0.27	-0.06	-0.08
M2	148	0.00	-	-	-	-	-	-
		0.10	0.24	-0.28	-0.29	0.21	0.01	-0.02
		0.20	0.44	-0.60	0.65	0.34	0.01	-0.04
		0.30	0.58	-0.96	-1.06	0.39	-0.01	-0.08
M3	153	0.00	-	-	-	-	-	-
		0.10	0.16	-0.22	-0.07	0.05	-0.06	0.02
		0.20	0.26	-0.49	-0.17	0.08	-0.16	0.00
		0.30	0.30	-0.82	-0.28	0.09	-0.31	-0.04

Table S2 – Lennard-Jones (LJ) interaction energy values between all species and the electrodes (in kcal/mol) for the models composed of a single hydrated ionic liquid (models M1 to M3).

Model	Ionic Pairs	σ (e/nm^2)	[bmim] – Graphene+	[bmim] – Graphene-	[Anion] – Graphene+	[Anion] – Graphene-	H_2O – Graphene+	H_2O – Graphene-
M1	152	0.00	-6.22	-6.18	-1.69	-1.66	-1.40	-1.44
		0.10	-5.59	-6.62	-1.89	-1.41	-1.70	-1.28
		0.20	-4.95	-7.03	-2.06	-1.13	-1.98	-1.12
		0.30	-4.19	-7.42	-2.21	-0.85	-2.32	-0.99
M2	148	0.00	-6.61	-6.68	-2.48	-2.53	-0.75	-0.69
		0.10	-6.20	-7.03	-2.80	-2.17	-0.80	-0.66
		0.20	-5.77	-7.31	-3.12	-1.76	-0.84	-0.68
		0.30	-5.17	-7.65	-3.33	-1.39	-1.03	-0.64
M3	153	0.00	-5.09	-5.14	-0.32	-0.33	-2.41	-2.38
		0.10	-4.47	-5.74	-0.37	-0.28	-2.75	-2.04
		0.20	-3.61	-6.35	-0.39	-0.24	-3.21	-1.71
		0.30	-2.83	-6.84	-0.42	-0.19	-3.62	-1.45

Table S3 – Coulomb interaction energy values between all species and the electrodes (in kcal/mol) for the models composed of a single hydrated ionic liquid (models M4 to M6).

Model	Ionic Pairs	σ (e/nm^2)	[bmim] – Graphene+	[bmim] – Graphene-	[ClO$_4$] – Graphene+	[ClO$_4$] – Graphene-	[Br] – Graphene+	[Br] – Graphene-	H_2O – Graphene+	H_2O – Graphene-
M4	150	0.00	-	-	-	-	-	-	-	-
		0.10	0.23	-0.27	-0.26	0.18	0.00	0.00	0.00	-0.01
		0.20	0.41	-0.58	-0.57	0.29	-0.01	0.01	-0.01	-0.04
		0.30	0.53	-0.93	-0.92	0.31	-0.02	0.01	-0.05	-0.07
M5	148	0.00	-	-	-	-	-	-	-	-
		0.10	0.23	-0.28	-0.21	0.15	-0.05	0.04	0.01	-0.02
		0.20	0.40	-0.59	-0.46	0.24	-0.12	0.06	-0.01	-0.04
		0.30	0.55	-0.94	-0.77	0.27	-0.22	0.07	-0.02	-0.09
M6	152	0.00	-	-	-	-	-	-	-	-
		0.10	0.19	-0.25	-0.02	0.01	-0.16	0.12	-0.02	-0.01
		0.20	0.35	-0.54	-0.03	0.02	-0.39	0.17	-0.05	-0.03
		0.30	0.39	-0.87	-0.06	0.02	-0.55	0.18	-0.13	-0.08

Table S4 – Lennard-Jones interaction energy values between all species and the electrodes (in kcal/mol) for the models composed of a single hydrated ionic liquid (models M4 to M6).

Model	Ionic Pairs	σ (e/nm^2)	[bmim] – Graphene+	[bmim] – Graphene-	[ClO_4] – Graphene+	[ClO_4] – Graphene-	[Br] – Graphene+	[Br] – Graphene-	H_2O – Graphene+	H_2O – Graphene-
M4	150	0.00	-6.55	-6.55	-2.29	-2.26	-0.02	-0.02	-0.82	-0.83
		0.10	-5.98	-6.74	-2.51	-1.81	-0.03	-0.03	-0.97	-0.90
		0.20	-5.43	-7.13	-2.73	-1.51	-0.03	-0.03	-1.14	-0.81
		0.30	-4.83	-7.42	-2.87	-1.12	-0.04	-0.03	-1.35	-0.81
M5	148	0.00	-6.50	-6.53	-1.80	-1.88	-0.44	-0.39	-0.96	-0.93
		0.10	-6.07	-6.93	-2.08	-1.58	-0.48	-0.36	-1.03	-0.85
		0.20	-5.39	-7.20	-2.19	-1.26	-0.53	-0.31	-1.31	-0.83
		0.30	-4.98	-7.51	-2.40	-0.94	-0.63	-0.24	-1.35	-0.84
M6	152	0.00	-5.92	-6.01	-0.07	-0.06	-1.27	-1.36	-1.69	-1.62
		0.10	-5.15	-6.28	-0.08	-0.06	-1.36	-1.07	-2.05	-1.52
		0.20	-4.78	-6.87	-0.07	-0.06	-1.69	-0.81	-2.17	-1.29
		0.30	-3.61	-7.08	-0.09	-0.05	-1.56	-0.58	-2.81	-1.22
